# Cracking in Sweet Cherry Cultivars Early Bigi and Lapins: Correlation with Quality Attributes

**DOI:** 10.3390/plants9111557

**Published:** 2020-11-12

**Authors:** Sandra Pereira, Vânia Silva, Eunice Bacelar, Francisco Guedes, Ana Paula Silva, Carlos Ribeiro, Berta Gonçalves

**Affiliations:** 1 CITAB-Centre for the Research and Technology of Agro-Environmental and Biological Sciences, University of Trás-os-Montes e Alto Douro, Quinta de Prados, 5000-801 Vila Real, Portugal; vaniasilva@utad.pt (V.S.); areale@utad.pt (E.B.); asilva@utad.pt (A.P.S.); bertag@utad.pt (B.G.); 2 Cermouros—Cerejas de São Martinho de Mouros, Lda, Quinta da Ribeira, Bulhos, 4660-210 Resende, Portugal; fjmguedes@cermouros.pt; 3 Department of Agronomy, University of Trás-os-Montes e Alto Douro, Quinta de Prados, 5000-801 Vila Real, Portugal; cribeiro@utad.pt

**Keywords:** cracking index, fruit quality, sweet cherry fruit, yield efficiency

## Abstract

Fruit cracking is one of the main concerns in sweet cherry production and is caused by a heavy rainfall before and during the harvest. This physiological disorder leads to severe economic losses, which can be more or less effective depending on the cracked region of the fruit: in the cheeks (side cracks), in the stylar scar region, or in the stem cavity region. Sweet cherry cracking can be affected by several factors such as cultivar, growing conditions, rootstock, fruit size, flesh osmotic potential, cuticular characteristics of the skin, and stage of fruit development. In this sense, the objective of this work was to evaluate the cracking incidence in two sweet cherry cultivars (Early Bigi and Lapins grafted on “Saint Lucie 64” rootstock) and correlate the cracking index with other quality parameters. Fruits were harvested on 2 May (cv. Early Bigi) and on 27 May (cv. Lapins) 2019 at their commercial ripening stage. In the field, the total yield and the trunk cross-sectional area were determined for each tree in order to calculate the yield efficiency. In the laboratory, the cracking index was determined in 150 fruits without visual defects. In addition, fruit size and weight, wax content, flesh firmness, epidermis rupture force, total soluble solids, pH, titratable acidity, and maturity index of 30 fruits were also evaluated. In general, all the analyzed quality parameters were influenced by the cultivar, being that cv. Lapins presented larger, heavier, firmer, and sweeter fruits, with more acidity and higher maturation index. However, cv. Lapins also presented higher cracking index, which was positively correlated with all the parameters above-mentioned and negatively correlated with the wax content. In fact, cv. Early Bigi presented a high wax content and simultaneously a low cracking index. The stylar scar region cracks were the most prevalent in both cultivars. These results allowed us to conclude that, in the North Portugal region, the Lapins cherries presented better quality attributes than the Early Bigi cherries. However, the latter are still very valuable to the region due to its early ripening. Additionally, it was also possible to conclude that bigger, firmer, more mature, and with lower wax content cherries were more sensitive to cracking than the smaller fruits, soft-fleshed, less mature, and with higher wax content.

## 1. Introduction

Sweet cherries *(Prunus avium* L.) are one of the most attractive and appreciated spring–summer fruits, especially due to its attractive appearance, color, taste, and sweetness [1].

According to FAOSTAT (Food and Agriculture Organization of the United Nations), in 2018, 2,547,944 tons of sweet cherry were produced in the world, in a global cultivated area of 432,314 ha [2]. In that year, the main producers in the world were Turkey, United States of America, Uzbekistan, Chile, and Iran [2]. Within Europe, Italy (114,798 tons), Spain (106,584 tons), Romania (90,837 tons), and Greece (90,290 tons) were the top sweet cherry producing countries, while Portugal produced, in the same year, 17,461 tons in a total cultivated area of 6056 ha [2].

Sweet cherries are extremely perishable fruits (short shelf life of 7–14 days in air cold storage) with a short harvest season. Its quality is highly affected by the environmental conditions, since excessive rainfall before and during the harvest can lead to the fruit cracking. This physiological disorder causes production and economic losses [3,4] and originates from the excess uptake of water by the fruit surface, which results in localized bursting of the skin [5,6]. Other authors support that fruit cracking is the result of skin shrinking after rapid cooling caused by rainfall or by a sharp temperature drop [7]. Cracking in sweet cherries can be divided in macro-cracks (extending into the epidermal and hypodermal cell layers and visible to the naked eye) and micro-cracks (induced by water and not detected by visual inspection) [6,8]. According to [9], three different types of macro-cracking can occur in sweet cherries: in the cheek region, in the stylar scar region (apical end), and in the stem cavity region.

The cracking susceptibility is difficult to quantify in the field, since the level, distribution, and duration of rainfall, fruit maturity stage, orchard factors, and environmental conditions are not standardized [10]. Indeed, fruit cracking can be affected by several factors such as cultivar, growing conditions, rootstock, fruit size, flesh osmotic potential, cuticular characteristics of the skin, and stage of fruit development [3,11]. Among the several factors affecting the cracking of sweet cherry, the effect of the cultivar on this disorder was evaluated in the present work. The cultivars studied were Early Bigi and Lapins.

The cv. Early Bigi, also known as Bigi Sol, is a self-sterile cherry cultivar, large in size but with less flavor than other cultivars such as Burlat and Lapins. However, it is of great agronomic interest, since it is one of the very early cultivars produced in Resende.

The cv. Lapins, also marketed as Cherokee, is a hybrid of Van (variety very used in cross pollinations for its excellent attributes) and Stella (first self-fertile variety) cultivars and it is currently the sweet cherry cultivar most planted around the world. This cultivar is a late-season sweet cherry cultivar, ripening about one month later than Early Bigi. Its fruit is of excellent quality, producing some of the largest and juiciest of the sweet cherries. Furthermore, this cultivar is self-fertile and do not require a pollinator.

The sensitivity of different cultivars to cracking is not completely understood. However, some authors support that by choosing the right cultivar, it is possible to minimize the cracking incidence. The authors in [12,13] also defended that good results in productivity, fruit size, firmness, and tolerance against cracking were the main criteria for cultivar selection.

In this sense, the main objective of this work was to evaluate the fruit cracking incidence and cracking characteristics of two sweet cherry cultivars Early Bigi and Lapins (early vs. late) and correlate it with other fruit quality parameters, namely, fruit size and weight, firmness, epidermis rupture force, wax content, total soluble solids, titratable acidity, maturity index, and pH.

## 2. Results and Discussion

### 2.1. Total Yield and Yield Efficiency

According to [14], high yield efficiency is an important characteristic that determines the commercial value of a given cultivar.

**Figure 1:**
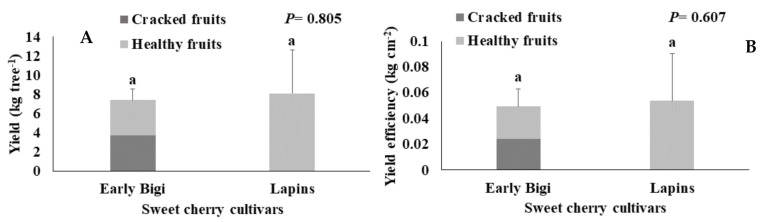
(**A**) Yield (kg tree^−1^) and (**B**) yield efficiency (kg cm^−2^) of two sweet cherry cultivars (Early Bigi. and Lapins). Data are expressed as mean ± standard deviation. Similar letters indicate no statistically significant differences (*p >* 0.05) between cultivars for each variable, according to Tukey’s test.

The results of the yield per tree evaluated in the field at harvesting time and the yield efficiency are presented in the Figure 1. No significant differences were observed in both parameters for the two studied cultivars (*p* = 0.805 and *p* = 0.657, respectively). Despite no significant differences were observed in the total yield per tree, 50.1% of the fruits of cv. Early Bigi were harvested already cracked, due to heavy rains during the maturity period (especially the month of April), while no cracked fruits were collected for cv. Lapins. This can be explained by the low rainfall during the month of May (only 0.3 mm of rain). In fact, high rainfall during the maturity period and near the harvest time usually leads to a higher cracking index.

The yield efficiency also did not present significant differences between cultivars. However, the highest values were observed in cv. Lapins. In a previous work developed by [13], in Slovenia, cv. Early Bigi presented a higher yield efficiency (0.25 kg cm^−2^) than our trees, which can be explained by the different ages of the trees, different soils, and edaphoclimatic conditions.

### 2.2. Cracking Index and Crack Type Incidence

**Figure 2:**
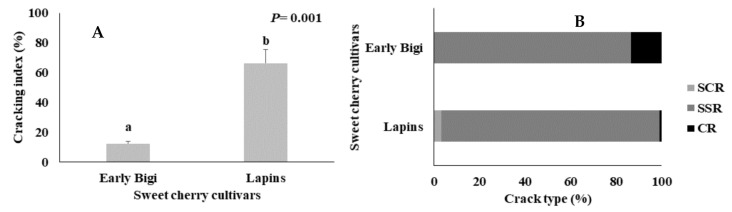
(**A**) Cracking index (%) and (**B**) crack type incidence (%) of two sweet cherry cultivars (Early Bigi and Lapins). SCR—stem cavity region, SSR—stylar scar region, and CR—cheek region. Data are expressed as mean ± standard deviation. Different letters indicate statistically significant differences (*p* < 0.05) between cultivars for each variable, according to Tukey’s test.

The CI (cracking index) of both sweet cherry cultivars after an immersion in distilled water during 6 h and the crack type incidence are presented in Figure 2. The cv. Lapins presented a significantly higher cracking index than cv. Early Bigi (*p =* 0.001), with a fold increase of 440%, despite being considered as a moderately resistant cultivar to cracking.

Taking into account the different types of cracking, in the present work, the SSR cracks were the most frequent in both cultivars (86.41% in cv. Early Bigi and 96.12% in cv. Lapins). This can be explained by the higher osmotic concentration of solutes in this part of the fruit, which accounted for a more rapid water absorption through the skin, resulting in a quicker formation of cracks [15]. Furthermore, cv. Early Bigi also presented 13.59% of CR cracks and no SCR-type cracking was found in this cultivar, while cv. Lapins presented 3.1% of SCR cracks and only around 0.78% of CR cracks. According to [15], the small cracks at the base and top end of the fruit (SCR and SSR cracks) often occurred at a very early stage, when the fruits were not yet mature. The same author [9] also suggested that cherries with these two types of cracks were tolerated by consumers since no fungal infection is present. However, fruit with CR cracks (more predominant in cv. Early Bigi than in cv. Lapins) are usually rejected by the consumers.

Despite the higher cracking index of cv. Lapins observed in the present work and determined in the laboratory, it is necessary to take in account that cracking of fruit attached to the tree and that of fruit detached and submerged differs significantly. In fact, according to [16], fruit attached to the tree cracked more slowly and required more water to crack.

### 2.3. Cracking Index and Biometric Attributes

Fruit size is considered as the main benchmark in commercial cherry grading, being a large factor in consumer preference, and is a huge determinant of both farm gate and market price.

**Figure 3:**
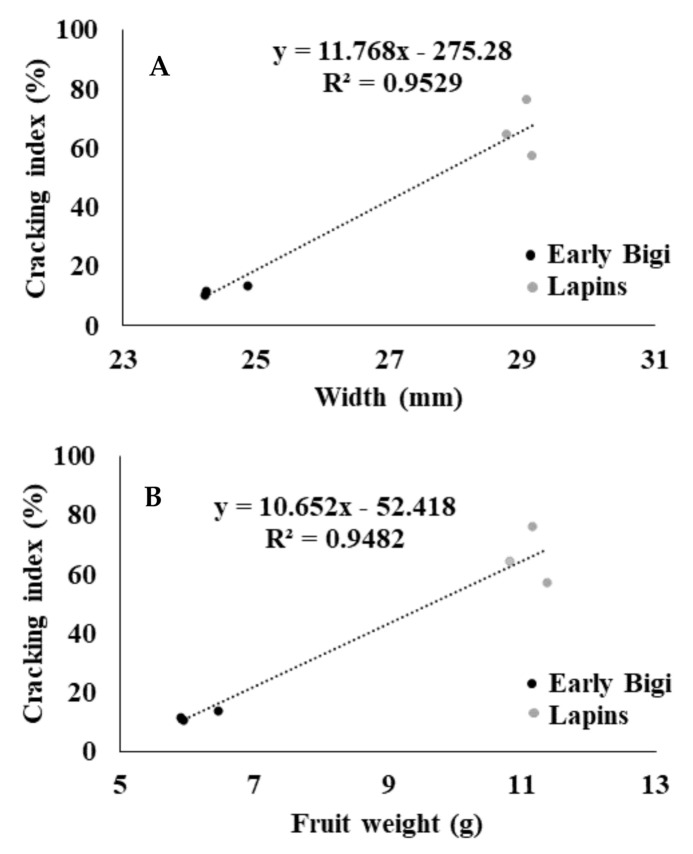
Correlation between (**A**) the cracking index (%) and the fruit width (mm) and (**B**) the cracking index (%) and the fruit weight (g) of two sweet cherry cultivars: Early Bigi and Lapins.

The correlations between the CI and the fruit width and its weight are presented in Figure 3A,B. In Figure 3A, it is possible to observe that cv. Early Bigi presented simultaneously lower values of CI and width, which means that these two parameters were positively correlated (R^2^ = 0.9529, *p* = 0.001). The same profile was observed in Figure 3B. Indeed, CI and fruit weight were also positively correlated (R^2^ = 0.9482, *p* = 0.001), with the highest values of both parameters to be observed in cv. Lapins. In fact, the weight of fruits from cv. Lapins was significantly higher (11.12 g of average weight) than in cv. Early Bigi (6.10 g of average weight) (*p* = 0.000). According to several authors, the fruit size depends on cultivar, maturation stage, agricultural practices, and environmental conditions [17,18,19]. In this work, the higher CI observed in cv. Lapins may have been influenced by the big fruit size of this cultivar (29.00 mm of average width) in contrast to cv. Early Bigi (24.46 mm of average width), thus there were significant differences between cultivars (*p* = 0.000). In fact, according to [20] and [21], larger fruits are more prone to cracking than small ones. In fact, in big fruits, the physical stress on the enclosing membrane (the skin) is bigger, and consequently, cracks occur in areas of the fruit where stress is the greatest [4]. However, the first author also suggested that the correlation between the CI and the fruit size was greater within a cultivar than between cultivars, since it is necessary to take into account the specificities of each cultivar.

### 2.4. Cracking Index, Soluble Cuticular Wax Content, and Texture Parameters

The fruit cuticle is a hydrophobic and semi-permeable membrane consisting of two major lipid types: cutin and cuticular waxes [22]. Cuticular waxes play an important role in the water permeability of sweet cherries [23].

**Figure 4:**
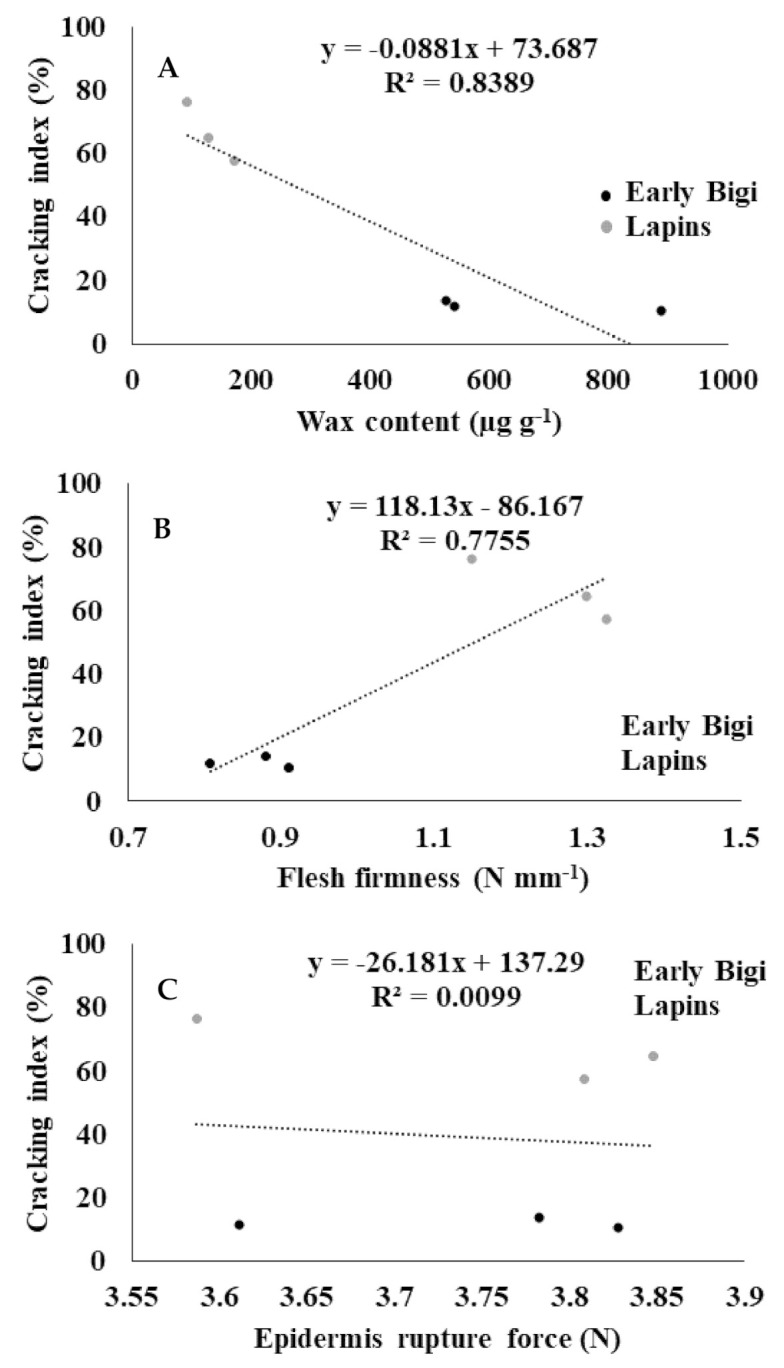
Correlations between (**A**) the cracking index (%) and the wax content (μg g^−1^), (**B**) the cracking index (%) and the flesh firmness (N mm^−1^), and (**C**) the cracking index (%) and the epidermis rupture force (N) of two sweet cherry cultivars: Early Bigi and Lapins.

The correlation between the CI and the cuticular wax content is presented in Figure 4A. Cv. Early Bigi presented lower CI, but significantly higher wax content than cv. Lapins (*p* = 0.000). This means that these two parameters were negatively correlated (R^2^ = 0.8389, *p* = 0.012). In general, the lower the wax content, the greater the water intake for the fruit, and consequently, the higher the cracking index. Indeed, according to [24], cultivars more tolerant to cracking have higher wax contents.

Fruit firmness (FF) is an important quality attribute in sweet cherries, which is associated with a greater resistance to decay and mechanical damage, and consequently, to the increase of storage life [25]. In the present work, according to Figure 4B, the CI is positively correlated with the FF (R^2^ = 0.7755, *p* = 0.021), being that cv. Lapins presented the highest values for both parameters. Indeed, cv. Lapins presented a significantly higher FF than cv. Early Bigi (*p* = 0.000). According to [26], sweet cherries with higher flesh firmness can have consequently fewer physiological disorders during handling, storage, and shipping, but according to [21], these firmer fruits are simultaneously more affected by cracking. In fact, it is usually assumed that cultivars with firm-fleshed fruits have more tendency to cracks, but the literature concerning this correlation is still very unclear [19]. It is likely that the epidermis elasticity is smaller in the hardest fruits.

In a previous work developed by [27,28], cv. Lapins presented an average fruit firmness of 2.65 N at harvest, which was lower than the results obtained in the present work for both cultivars (3.75 N for Lapins and 3.74 N for Early Bigi), perhaps due to the pre-harvest applied treatments and/or the different edaphoclimatic conditions.

No correlation was observed between the CI and the epidermis rupture force (ERF) (R^2^ = 0.0099, *p* = 0.870) (Figure 4C). Furthermore, no significant differences were observed in ERF between cultivars (*p* = 0.963).

### 2.5. Cracking Index (CI) and Maturity Evaluation Parameters

**Figure 5:**
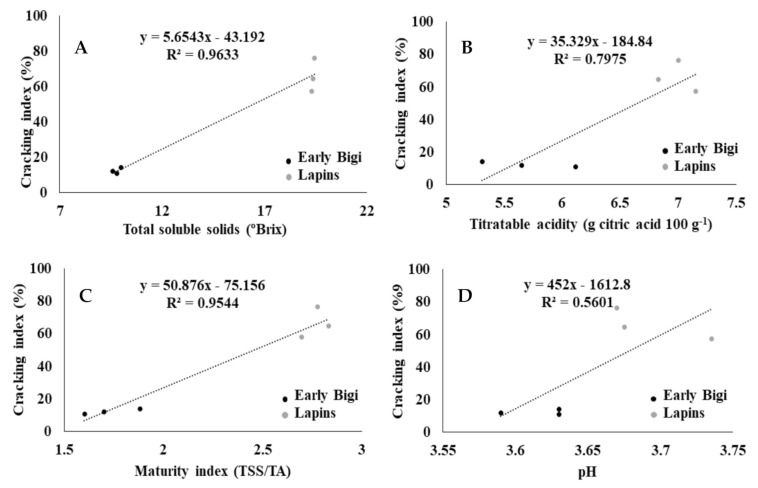
Correlations between (**A**) the cracking index (%) and the total soluble solids (°Brix), (**B**) the cracking index (%) and the titratable acidity (g citric acid 100 g^−1^), (**C**) the cracking index (%) and the maturity index (TSS/TA), and (**D**) the cracking index (%) and the pH of two sweet cherry cultivars: Early Bigi and Lapins.

The cv. Lapins presented higher values for all the analyzed routine parameters (Figure 5A–D). According to [29], total soluble solids (TSS) depend on cultivar, mainly due to glucose and fructose and less to the presence of sucrose and sorbitol, being related to flavor intensity. In this work, cv. Lapins presented a TSS value twice higher than that of cv. Early Bigi (averages of 19.37 and 9.80 °Brix, respectively), indicating a significantly higher sugar content in cv. Lapins (*p* = 0.000). Maturity index (MI), as the ratio between TSS and TA (titratable acidity), is also an indicator of sweetness and consequently of maturity [30,31], allowing us to conclude that cv. Lapins (MI = 2.77) was significantly more mature at harvesting time than cv. Early Bigi (MI = 1.73) (*p* = 0.000). As cv. Early Bigi is one of the earliest cultivars collected in the Resende region, it can be sold at a good price and it is therefore a very profitable cultivar in the region.

Positive correlations were observed between the CI and all the analyzed routine parameters, with the exception of pH (Figure 5A–D). This means that the higher the total soluble solids, the titratable acidity and the maturity index, the greater the CI. Stronger correlations were found between the CI and TSS (R^2^ = 0.9633, *p* = 0.001) and the CI and the MI (R^2^ = 0.9544, *p* = 0.001). These results were consistent with the findings of [32], who also reported a positive correlation between the TSS and fruit cracking in sweet cherry cultivars. [33] also demonstrated that the susceptibility to cracking increased with the maturation stage.

### 2.6. Principal Component Analysis (PCA)

**Figure 6:**
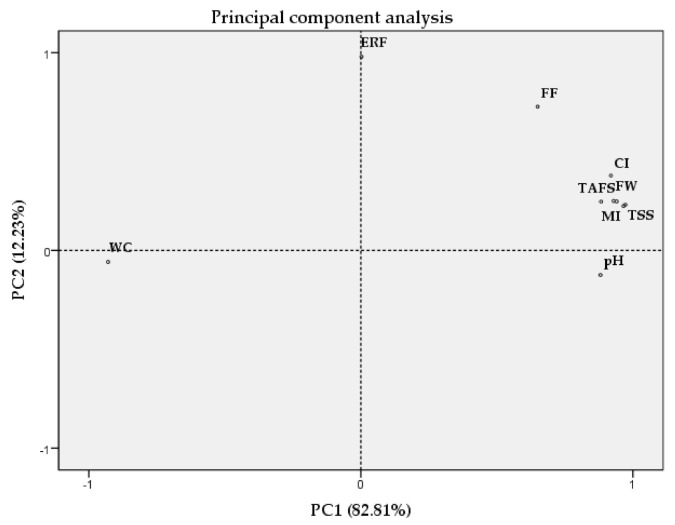
Principal component analysis using the whole dataset of two sweet cherry cultivars (Early Bigi and Lapins). Analyzed parameters: WC—wax content; ERF—epidermis rupture force; FF—flesh firmness; pH; CI—cracking index; FW—fruit weight; FS—fruit size (width); TA—titratable acidity; TSS—total soluble solids; MI—maturity index.

To better understand the correlations between all the evaluated parameters, a chemometric analysis was performed integrating all the data (Figure 6). The PCA based on the correlation matrix standardizes the data and this analysis was performed using a correlation matrix (Corr-PCA). In this analysis, the first two factorial axes (PC1 and PC2) represent 95.04% of the total variance, with factor 1 the one that presents the higher weight (82.81%).

The CI as well as the most of the analyzed parameters (fruit size, fruit weight, total soluble solids, titratable acidity, and maturity index) were together in the right PCA quadrant as well as flesh firmness, epidermis rupture force, and pH, although the latter were further away from the remaining parameters. On the other hand, the wax content was spatially separated and placed in the left PCA quadrant, corroborating the negative correlation between this parameter and the CI.

## 3. Material and Methods

### 3.1. Experimental Design and Sweet Cherry Raw Material

The experiment was conducted in 2019 in a 10 year-old sweet cherry orchard located in São João de Fontoura, Resende (Viseu district, Northern Portugal; latitude 41°12′ N, longitude 7°93′ W, altitude 149 m).

Weather data were recorded by a weather station located near the experimental site. The daily minimum temperature in March, when the first flowers appeared in the trees, ranged between 3.7 and 12.2 °C and the daily maximum temperature ranged between 11.9 and 22.6 °C. The total precipitation in that month was 4.1 mm, with day 6 as the rainiest. April was a very rainy month, with a total rainfall of 8.1 mm throughout the month. The daily minimum temperature ranged between 2.0 and 14.3 °C and the daily maximum temperature varied between 10.9 and 26.0 °C. The hottest days were 29 and 30 April. May was the warmest month (daily minimum temperature ranged between 6.4 and 17.4 °C and daily maximum temperature fluctuated between 16.2 and 32.9 °C) and the least rainy month (only 0.3 mm of total precipitation).

For this study, ten trees of each sweet cherry cultivar (Early Bigi and Lapins, grafted on ‘Saint Lucie 64′ rootstock) were used. Trees were spaced 3.0 m between rows and 2.5 m from each other in the row. Fruits were hand-picked at their commercial ripening stage, which was on 2 May and 27 May 2019 for cv. Early Bigi and cv. Lapins, respectively. The commercial ripening stage for each cultivar was defined by the producer. In fact, the fruits to analyze were collected at the same time that the producer harvested it to send to the market. Within each cultivar, about 500 g samples of fruit were collected randomly from all trees and two fruits per tree were additionally collected to determine the wax content. In the field, collected fruits were kept on ice and placed in the refrigerator upon reaching the laboratory. There, fruits were randomly divided into two sub batches. The first sub batch (150 sweet cherries without visual defects) was assigned to the evaluation of the cracking index (CI), while the second sub batch, consisting of 30 sweet cherries, was assigned for fruit weight (FW), size (FS), epidermis rupture force (ERF), flesh firmness (FF), total soluble solids (TSS), titratable acidity (TA), maturity index (MI), and pH measurements.

### 3.2. Production and Yield Efficiency

At harvest time, the production per tree was assessed, evaluating the weight of healthy and cracked cherries, separately. The perimeter of the trunks was also measured to determine the trunk cross-sectional area (TCSA) and the yield efficiency was determined as the ratio between the yield and the TCSA (kg cm^−2^).

### 3.3. Cracking Index

The cracking index (CI) was determined in the first sub batch of cherries according to the method of [34], and modified by [15]. For this, the 150 cherries were divided into three replicates of 50 fruits and immersed in 2 L containers filled with distilled water (20 ± 1 °C). After 2, 4, and 6 h, the fruits were observed for macroscopic cracks. At each time, cracked cherries were removed and counted, and fruits without cracks were re-incubated. The CI was calculated as follows:

CI = ((5*a* + 3*b* + *c*) ∗ 100)/250 (1)

where *a*, *b*, and *c* represent the number of cracked cherries after 2, 4, and 6 h of immersion, respectively.

The crack type (CT) incidence (%) was also determined and expressed as the number of cracks of a particular type (SCR—stem cavity region, SSR—stylar scar region or CR—cheek region) compared to the total number of cracks.

### 3.4. Soluble Cuticular Waxes Content

Total soluble epi- and intra-cuticular wax content of sweet cherries was determined using two fruits per tree, by the method of [35]. Two fruits without peduncle per tree were weighed and immersed in a mix of chloroform:methanol (3:1) for 2 min at 25 °C. After that, the fruits were discarded and the solution was filtered and placed in a glass previously identified and weighed. After about a week, when the glasses were dry, they were weighted again. The weight of the fruits and the difference in the weight of the glasses were used to calculate the wax content, which was expressed as μg g^−1^ of fresh weight.

### 3.5. Fruit Weight and Size

Fruit weight and size were determined for the 30 fruits of the second sub batch. Fruit weight (g) was evaluated using an electronic balance (EW2200-2NM, Kern, Germany) and fruit size was determined by measuring the width (mm) using electronic calipers (500-196-30, Mitutoyo, UK).

### 3.6. Epidermis Rupture Force and Flesh Firmness

The same 30 fruits from the second sub batch were used to determine the texture parameters, epidermis rupture force (N) and flesh firmness (N mm^−1^) using a TA.XT.plus texture analyzer (Stable Micro Systems, Godalming, UK) employing a 50 N loading cell and a 2.0 mm diameter cylindrical probe. The maximum force when compressing 5 mm was measured at a speed of 1 mm s^−1^.

### 3.7. Total Soluble Solids, Titratable Acidity, Maturity Index, and pH

After determining the texture, the same 30 fruits from the second sub batch were divided into three groups of 10 fruits. The juice from these 10 fruits was extracted with an electrical extractor (ZN350C70, Tefal Elea, China) for one minute. Total soluble solids (TSS, in °Brix) were determined using a digital refractometer (PR-101, Atago, Tokyo, Japan) and expressed as the percentage of soluble solids in juice (%). Then, pH was also measured using a pH meter (3310 Jenway). Titratable acidity (TA, in g citric acid 100 g^−1^ of fresh weight) was determined by diluting 10 mL of fruit juice with 10 mL of distilled water and titrating with 0.1 mol L^−1^ sodium hydroxide (NaHO) until reaching pH 8.2 using a Schott Easy Titroline automatic titrator. The maturity index was expressed as the ratio of TSS and TA.

### 3.8. Statistical Analysis

Statistical analysis was performed using SPSS V.25 software (SPSS-IBM, Corp., Armonk, New York, NY, USA). Statistical differences between the two cultivars were evaluated by one-way analysis of variance (ANOVA), followed by Tukey’s post-hoc multiple range test (*p <* 0.05). In addition, Pearson’s rank correlation was performed for the relationship between the cracking index and the other quality parameters (three average values for each cultivar). Principal component analysis (PCA) was also performed. PCA is mostly used as a tool in exploratory data analysis and for making predictive models. PCA was performed by eigenvalue decomposition of the data correlation (Corr-PCA) matrix after normalizing the data matrix for each attribute.

## 4. Conclusions

Nowadays, the big challenge of producers is to guarantee high production without affecting the quality of the fruit. For this, the selection of the best cultivars for each region is crucial.

In the present work, cv. Lapins cherries presented better quality attributes than the cv. Early Bigi cherries, having potentially greater commercial value and is an ideal cultivar for the Resende region. Lapins cherries simultaneously presented a higher cracking index, which was related to the bigger size, higher fruit firmness, more mature fruits, and less wax content. However, it is necessary to take into account that the cracking index is higher in detached fruits compared to cracking in fruits on the trees. Even so, Lapins cherries presented higher susceptibility for cracking in the laboratory assays, indicating a possible higher cracking incidence in the orchard if intensive rains occur near or during the harvest.

Although it has not shown itself as an ideal cultivar in terms of quality parameters, the cv. Early Bigi is still very valuable to producers in the Resende region due to its early ripening. The differences in terms of productivity and harvest time of these two cultivars are also important to the producers, especially due to the climate change and irregular rainfall that can affect one cultivar more than the other, according to its maturity stage.
